# Unveiling Gait Variability Post-ACLR: Using Lyapunov Exponent and Force-Sensing Insoles

**DOI:** 10.51894/001c.143953

**Published:** 2025-09-30

**Authors:** Chaitanya Manam

**Affiliations:** 1 College of Osteopathic Medicine Michigan State University

## 30

### INTRODUCTION

Following anterior cruciate ligament reconstruction (ACLR), gait biomechanics often change, increasing the risk of early osteoarthritis (EOA). Ground reaction force (GRF) variability during gait may contribute to EOA development by altering knee joint loading. Traditional biomechanical assessments overlook how variance evolves over time, relying on short trials and standard deviation as a measure of variance. Therefore, examining gait variability over longer periods using the largest Lyapunov exponent (LLE) and accessible force-sensing insoles is necessary.

### OBJECTIVE

To examine differences in GRF variability between (1) injured and contralateral limbs and (2) individuals with and without EOA.

### METHODS

Thirty-four participants (13 females, 34.2%; age: 19.9 ± 8.1 years; height: 169.5 ± 9.6 cm; mass: 70.7 ± 14.1 kg; time since surgery: 6.3 ± 1.2 months) post primary unilateral ACLR were included. EOA status was determined using Englund et al.’s criterion based on the patient reported outcome survey: Knee Injury and Osteoarthritis Outcome Score. GRF was measured using force-sensing insoles during a 30-second treadmill trial at a self-selected speed. GRF variability was assessed in both limbs using LLE, a unitless measure where greater values above zero indicate greater variability/chaos. LLE was calculated across all steps with phase space reconstruction parameters (embedding dimensions: 3-5, lags: 1-10). A two-way mixed ANOVA compared LLE across EOA status and limb.

### RESULTS

Sixteen participants met EOA criteria. Mean LLE for the healthy limb was 0.16 ± 0.01, while the ACLR limb was 0.14 ± 0.01. A two-way mixed ANOVA revealed no significant effects of EOA status (p = 0.68) or limb (ACLR vs. contralateral, p > 0.99) on LLE values, nor a significant interaction (p = 0.35).

### CONCLUSION

GRF variability was similar between limbs and between individuals with and without EOA. However, LLE values suggest chaotic GRF patterns in both limbs during treadmill gait. Longitudinal studies should track LLE changes over time, particularly from early to later postoperative stages. Future research should include a healthy control group to determine whether observed variability reflects an adaptive ACLR response.

